# Toxic epidermal necrolysis and Stevens‐Johnson syndrome after COVID‐19 infection and vaccination

**DOI:** 10.1111/ajd.13958

**Published:** 2022-12-09

**Authors:** Henry Zou, Steven Daveluy

**Affiliations:** ^1^ Michigan State University College of Human Medicine Grand Rapids Michigan USA; ^2^ Department of Dermatology Wayne State University School of Medicine Detroit Michigan USA

**Keywords:** COVID‐19, keratinocyte, Stevens‐Johnson syndrome (SJS), T‐cell, toxic epidermal necrolysis (TEN)

## Abstract

Stevens‐Johnson Syndrome (SJS) is a rare but severe skin reaction characterized by blistering and peeling of the skin and ulcerations of mucous membranes; toxic epidermal necrolysis (TEN) is a subset of SJS characterized by the involvement of >30% of the skin. Though previously associated with drugs and infections, discussions on the association between TEN/SJS and COVID‐19 have been limited. We present a review of TEN/SJS after COVID‐19 infection and vaccination. Literature searches were conducted on PubMed and Google Scholar from 2019 to 8/2022. Thirty‐eight articles were selected based on subject relevance, and references within selected articles were also screened for relevance. As of 8/2022, there have been 34 published cases of TEN, SJS, and SJS‐TEN overlap after COVID‐19 infection and vaccination, including 12 cases after vaccination and 22 cases after infection. Multiple authors hypothesize that virotopes or excipients in COVID‐19 vaccines can activate T‐cells or cytokines to induce TEN/SJS. Meanwhile, some hypothesize that COVID‐19 infection induces immune activation that can trigger TEN/SJS or increase susceptibility to drug‐induced TEN/SJS. Treatments for post‐infection and post‐vaccination TEN/SJS vary significantly. We recommend remaining vigilant for this rare and severe potential complication.

## INTRODUCTION

Stevens‐Johnson syndrome (SJS) describes a rare yet severe immune‐mediated skin disorder characterized by blistering, detachment of the epidermis forming painful raw areas and ulceration of the mucous membranes that affect <10% of the total body surface area (BSA).^1^ Toxic Epidermal Necrolysis (TEN) represents a more extensive form of Stevens‐Johnson Syndrome (SJS) in which >30% of the BSA is involved.^1^ Meanwhile, epidermal involvement between 10 and 30% of the BSA is considered SJS‐TEN overlap.^1^, ^2^ TEN/SJS pathophysiology is not fully elucidated but is known to involve a type IV hypersensitivity reaction in which activated cytotoxic T‐cells release granulysin and cytokines such as interleukin‐15 (IL‐15) to induce keratinocyte apoptosis.^1^ TEN/SJS incidence was reported to be 1.6 cases among 1 million US adults from 2009 to 2012.^3^ Both SJS and TEN can mediate life‐threatening septic and hypovolemic shock and the US mortality rate for TEN/SJS range from 19.4 to 29%.^1^, ^3^ TEN/SJS is typically associated with drugs including antibiotics, antiepileptics, non‐steroidal anti‐inflammatory drugs and immune checkpoint inhibitors.^1^ More rarely, it has also been associated with viruses (influenza, Epstein–Barr, Coxsackie, cytomegalovirus, parvovirus, human herpes virus 6 and 7), bacteria (Group A streptococcus, mycoplasma pneumonia), and in very rare cases malignancy and vaccines (influenza, tetanus, smallpox, varicella, anthrax).^1^, ^4^ However, discussion on the potential association between TEN/SJS and COVID‐19 remains limited. We present an updated review of TEN/SJS onset after COVID‐19 infection and vaccination and its implications for adverse effects monitoring.

## METHODS

Literature searches were conducted on MEDLINE (PubMed), Scopus, Embase and Google Scholar ranging from 2019 to 8/2022. In total, 38 articles were selected based on subject relevance, and references within those articles were also screened. Articles reporting non‐TEN/SJS cutaneous manifestations that resembled TEN/SJS were excluded. Cases involving <10% of body surface area (BSA) were considered SJS, between 10 and 30% of BSA were considered SJS‐TEN, and >30% of BSA were considered TEN.

## RESULTS

**FIGURE 1:**
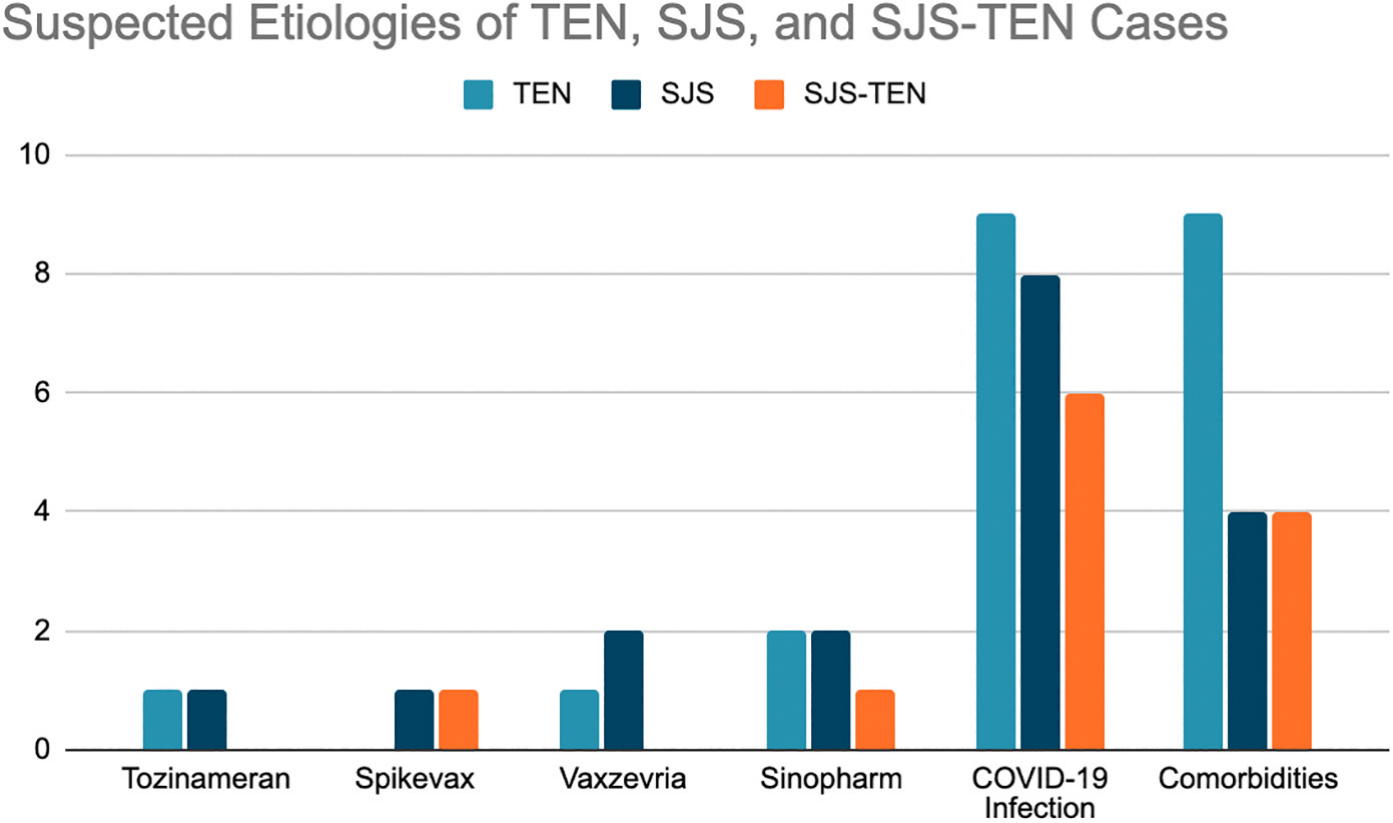
Graph of TEN, SJS‐TEN, and SJS cases after COVID‐19 infection and vaccination

To date (8/2022), there have been 34 published TEN, SJS and SJS‐TEN cases after COVID‐19 infection and vaccination. Twelve cases followed COVID‐19 vaccination (M_age_ = 56.90 years, R_age_ = 32–92 years, Male:Female:unspecified gender = 5:6:1). Twenty‐two cases followed COVID‐19 infection (M_age_ = 47.19 years, R_age_ = 6–81 years, Male:Female = 8:14) (Table 1). Fourteen COVID‐19 infection cases were diagnosed using real‐time reverse transcription polymerase chain reaction (rRT‐PCR), two cases tested negative on rRT‐PCR but COVID‐19 infection was nonetheless suspected based on chest X‐ray (CXR) findings and seven cases were diagnosed using unspecified COVID‐19 tests. None of the cases was flares of existing SJS/TEN. Tozinameran (Pfizer‐BioNTech) was linked to 2 cases, Spikevax (Moderna) to 2 cases, Vaxzevria (Oxford‐AstraZeneca) to 3 cases and Sinopharm to 5 cases. There have not been any cases linked to Jcovden (Johnson & Johnson) in the literature thus far, but the possibility of underreporting as a confounding factor cannot be ruled out. Comorbid diseases and medication use were linked to 17 cases (Figure 1).

**TABLE 1:** Cases of TEN, SJS‐TEN, and SJS after COVID‐19 infection and vaccination

Patient age and gender	Classification by BSA	Infection or vaccination?	COVID‐19 vaccine type	Comorbidities	Latency (days)	Distribution	Treatment	Outcome
49‐y.o. F^2^ (Bakir et al.)	TEN (>30% BSA)	Vaccination	Tozinameran, first dose	N/A	7 days	Trunk, face, upper limbs, oral mucosa	Etanercept 50 mg/mL subcutaneously, two doses spaced over days	No new lesions after the first dose, complete healing after 22 days
46‐y.o. F^14^ (Padniewski et al.)	SJS‐TEN (20% BSA)	Vaccination	Spikevax, first dose	N/A	1 day	Eyelids, cheeks, oral and nasal mucosa, arms, trunk, palms, soles	Prednisone 80 mg daily and clobetasol 0.05% ointment BID	Improved with therapy, discharged after 6 days of hospitalization
65‐y.o. M^9^ (Aimo et al.)	SJS (9% BSA)	Vaccination	Vaxzevria, second dose	N/A	10 days	Face, trunk, limbs, labial mucosa, glans penis	Prednisone 1 mg/kg/day tapered × 8 weeks	Progressive improvement to complete resolution
‘Middle‐aged’ F^25^ (Elboraey and Essa)	SJS (<10% BSA)	Vaccination	Tozinameran, second dose	N/A	5 days	Buccal and labial mucosa, tongue, palate, lips	Oral prednisolone (30 mg/day), triamcinolone acetonide and saline mouthwash	Unspecified
49‐y.o. F^16^ (Mansouri et al.)	SJS (<10% BSA)	Vaccination	Sinopharm, second dose	N/A	3 days	Lips, oral cavity, vagina, left palm	Fexofenadine 180 mg daily prednisolone 30 mg daily tapered × 3 weeks	Marked resolution of lesions after 2 weeks
32‐y.o. M^15^ (Boualila et al.) *Ophthalmic‐focused	SJS (<10% BSA)	Vaccination	Sinopharm, second dose	N/A	6 hours	Hands, chest, back, scalp, face, neck, conjunctiva and corneas	Eyewash, corticosteroid and tobramycin eye drops and ointment, oral vitamins A & C, ointment, symblepharon ring, oral doxycycline, artificial tears	Regression of palpebral, conjunctival and corneal symptoms after 30 days. Development of ectropion in the left eye.
92‐y.o. M^10^ (Seck et al.)	TEN (30–40% BSA)	Vaccination	Sinopharm, first dose	Trimethoprim‐sulfamethoxazole use 24 h before the onset of symptoms	20 days	Back and upper limbs	Fluid and electrolyte balance, nutritional support, pain management	Died 5 days after admission
60‐y.o. M^11^ (Dash et al.)	SJS (<10% BSA)	Vaccination	Vaxzevria, first dose	N/A	3 days	Trunk, arms, face, orolabial mucosa, eyes	Cyclosporine 300 mg PO, defer second vaccine dose	Complete resolution after 7 days
46‐y.o. unspecified gender^26^ (McMahon et al.)	SJS (<10% BSA)	Vaccination	Spikevax, first dose	Unspecified	Unspecified	Generalized, oral and genital mucosa	Unspecified	Unspecified
76‐y.o. M^12^ (Mardani et al.)	TEN (42% BSA)	Vaccination	Sinopharm, first dose	N/A	1 day	Face, trunk, upper and lower limbs, conjunctiva, oral mucosa, lips	Prednisolone, diphenhydramine‐lidocaine‐aluminium‐magnesium mouthwash with nystatin oral suspension, clotrimazole/triamcinolone/mupirocin combination ointment for lips	Discharged after 11 days, resolution after 2 weeks
48‐y.o. F^13^ (Kherlopian et al.)	TEN (90% BSA)	Vaccination	Vaxzevria, first dose	N/A	14 days	Trunk, limbs, orogenital mucosa	Adalimumab 80 mg subcutaneous injection, three doses over 5 days	Full recovery, discharged after 55 days
63‐y.o. F^27^ (Mansouri and Farshi)	SJS‐TEN (20% BSA)	Vaccination	Sinopharm, first dose	N/A	1 day	Oral mucosa, upper lip, trunk, extremities	Oral prednisolone 40 mg/day, cetirizine 10 mg BID, clobetasol propionate 0.05% ointment	Regression after 2 days, oral and lip lesions healed after 1 week, complete resolution after 2 weeks
23‐y.o. M^28^ (Abdelgabar and Elsayed)	SJS‐EM overlap (<10% BSA)	Infection (per rRT‐PCR)	N/A	N/A	14 days	Oral mucosa, arms, legs, glans penis	IV fluids and analgesia	Good recovery with complete resolution
43‐y.o. M^29^ (Hasani et al.)	SJS‐TEN (18% BSA)	Infection (per rRT‐PCR)	N/A	N/A	3–4 days	Face, fingers, mouth, genital area	IVIG 1 g/kg/day (IV dexamethasone 8 mg BID and remdesivir infusions for COVID‐19)	Bilateral central retinal vein occlusion on day 2 of admission, died of cardiopulmonary failure on day 6 of admission
57‐y.o. F^17^ (Pudukadan and John)	TEN (>30% BSA)	Infection (per rRT‐PCR)	N/A	N/A	13 days	Lips, conjunctiva, buccal and genital mucosa, face, neck, upper and lower limbs	Cyclosporine 100 mg TID tapered × 14 days, steroid‐antibiotic and lubricant eye drops	Resolution without recurrence after 1 month
48‐y.o. F^18^ (Aulakh et al.)	SJS‐TEN (>10% BSA)	Infection (suspected per CXR findings)	N/A	Paracetamol and allopurinol use × 2d	10 days	Trunk, bilateral limbs, lips, buccal and genital mucosa, conjunctiva	Unspecified	Unspecified
60‐y.o. F^18^ (Aulakh et al.)	SJS‐TEN (>10% BSA)	Infection (suspected per CXR findings)	N/A	Paracetamol and diclofenac × 3d	20 days	Lips, vulval and conjunctival mucosa, legs, flanks, bilateral breasts	Unspecified	Unspecified
77‐y.o. M^30^ (Manciuc et al.)	SJS (<10% BSA)	Infection (per rRT‐PCR)	N/A	Allopurinol use	14 days	Chest, back, upper and lower limbs, scalp, forehead	Methylprednisolone 250 mg/day, 20 mg bilastine BID, IV vitamin C 500 mg BID, gluconic calcium 10 ml/day, vitonal and gentamicin cream BID, cream of 5 g urea/1 g hydrocortisone/100 g petroleum jelly BID, meropenem 4 mg/day, linezolid 2 g/day, enoxaparine sodium 0.6 mg/day, acetaminophen 500 mg, acetylcysteine 600 mg/day	Most lesions above the legs healed after 5 days, but the patient suffered respiratory decompensation on day 7 and entered metabolic acidosis. Died after 20 days of hospitalization.
Unspecified‐y.o. F^31^ (Bitar et al.)	SJS (<10% BSA)	Infection (unspecified diagnostic test)	N/A	N/A	Unspecified	Back	Unspecified	Unspecified
58‐y.o. F^6^ (Lagziel et al.)	SJS‐TEN (5% BSA)	Infection (per rRT‐PCR)	N/A	Imatinib, vancomycin, piperacillin and tazobactam use	21 days	Thighs, arms and face	D/c antibiotics, hydrocortisone prophylaxis. Silver antimicrobial foam dressing changed twice weekly, oral prednisone tapered × 1 week	Discharged to a nursing facility, where symptoms resolved after completing the predisone taper
75‐y.o. F^32^ (Muhd Besari et al.)	SJS (<10% BSA)	Infection (unspecified diagnostic test)	N/A	N/A	9 days	Oral mucosa, trunk, conjunctiva, genitals	Supportive care	Complete resolution after 1 month
6‐.yo. M^22^ (Varol et al.)	TEN (32.5%)	Infection (per rRT‐PCR)	N/A	Influenza A	21 days	Face, chest, abdomen, genitals, upper limbs	IVIG (2 g/kg) and methylprednisolone (2 mg/kg/day), then three sessions of plasmapheresis 4 days later with pulse steroid and IVIG after each session	Discharged after 12 days of hospitalization with good healing but rudimentary scar tissue
6‐y.o. F^22^ (Varol et al.)	TEN (44.5% BSA)	Infection (per rRT‐PCR)	N/A	Augmentin use	1 day	Face, trunk, distal extremities, oral mucosa	Methylprednisolone (2 mg/kg/day), IVIG (2 g/kg), whole‐body debridements, synthetic absorbable microcell dressing (Suprathel), supportive care	Discharged after 21 days of hospitalization with complete skin integrity
6‐y.o. M^33^ (Jouhar et al.)	TEN (>30% BSA)	Infection (per rRT‐PCR)	N/A	Ibuprofen use	12 days	Oral mucosa, conjunctiva, trunk, genitals, palms, and soles	IVIG 1 g/kg daily × 5 days, IV dexamethasone later changed to oral prednisolone, and cyclosporin 3 mg/kg/day	Improvement in 1 week and discharged in stable condition. Recovery of the skin and mucosa in 1 month.
7‐y.o. M^34^ (Metbulut et al.)	SJS (<10% BSA)	Infection (per rRT‐PCR)	N/A	Augmentin use	2 days	Face, scalp, neck, trunk, oral and anal mucosa	Unspecified	Rash subsided in 14 days, but he died with 2° pulmonary involvement
78‐y.o. F^20^ (Rossi et al.)	TEN (~70% BSA)	Infection (unspecified diagnostic test)	N/A	Hydroxychloroquine use	~18 days	Flexural folds, trunk, buttocks, buccal and nasal mucosa	Methylprednisolone 1 mg/kg and IVIG 1 mg/kg × 3 days, then oral prednisone 1 mg/kg daily tapered over 1 month	Progressive improvement and eventual complete resolution over 6 weeks
62‐y.o. M^7^ (Saha et al.)	TEN (>30% BSA)	Infection (unspecified diagnostic test)	N/A	Allopurinol/Cotrimoxazole/Lenalidomide use	42 days	Palms and soles, oral mucosa, abdomen	IVIG 2 g/kg × 3 days	Rapid attenuation of TEN
53‐y.o. F^35^ (Narang et al.)	SJS‐TEN (>10% BSA)	Infection (per rRT‐PCR)	N/A	Metastatic breast carcinoma with bone, brain, and liver metastases	5 days	Chest, back, arms, legs, scalp, ears, and mouth	Conservative supportive/palliative care (2° metastatic malignancies)	Slow improvement
8‐y.o. M^36^ (Parlakay et al.)	SJS (<10% BSA)	Infection (unspecified diagnostic test)	N/A	Amoxicillin/Clavulanate use	1 day	Trunk	Prednisolone (2 mg/kg) and IVIG (0.5 g/kg)	Rashes responded well, but respiratory distress and pericardic infiltration worsened until treated with azithromycin and hydroxychloroquine
42‐y.o. F^5^ (Davoodi et al.)	SJS (<10% BSA)	Infection (per rRT‐PCR)	N/A	Hydroxychloroquine use × 2d	4 days	Generalized, orolabial and genital mucosa	Loratadine 10 mg BID, diphenhydramine 50 mg TID	Discharged after 5 days with nonpruritic scalded skin on distal upper extremities
50‐y.o. M^37^ (Pagh and Rossau)	SJS (<10% BSA)	Infection (per rRT‐PCR)	N/A	N/A	“A few days”	Lips, tongue, buccal mucosa, conjunctiva	Prednisolone 25 mg PO, moisturizing lip care	Symptom relief, discharged, resolution on f/u
76‐y.o. F^24^ (Krajewski et al.)	TEN (70% BSA)	Infection (unspecified diagnostic test)	N/A	Metamizole injection 2 days prior	2 days	Trunk, oral and vaginalmucosa	Daily TPE (5 cycles) and 90 g IVIG infusion	Lesions stabilized after 7 days, discharged with skin nearly fully healed after 14 days
45‐y.o. F^38^ (Shahraki et al.) *Ophthalmic‐focused	SJS (<10% BSA)	Infection (per rRT‐PCR)	N/A	Azithromycin and naproxen use × 3d	3 days	Conjunctiva, trunk, extremities	Topical levofloxacin, topical methylprednisolone 1%, frequent lubrication, autologous serum. q6h 1 week later	Recovery after. 3 weeks, but residual right superotemporal corneal scar, meibomian gland dysfunction and bilateral irregular eyelid margins
81‐y.o. F^19^ (Tanaka et al.)	TEN (>30% BSA)	Infection (per rRT‐PCR)	N/A	Hydroxychloroquine use	40 days	Trunk	Prednisolone 0.6 mg/kg/day tapered × 16 days	Improvement of symptoms
30‐y.o. F^21^ (Emadi et al.)	TEN (40% BSA)	Infection (per rRT‐PCR)	N/A	Phenobarbital use × 2 weeks	3 days	Back, upper limbs, conjunctiva, lips	Prednisolone 50 mg/day and IVIG 3 g/kg × 4 days, then cyclosporine 4 mg/kg	Progression stopped after 5 days, satisfactory recovery on cyclosporine treatment

Abbreviations: BID, twice daily; BSA, Body surface area; CXR, chest X‐ray; d, day(s); D/c, Discontinued; EM, erythema multiforme; f/u, follow‐up; IV, intravenous; IVIG, intravenous immunoglobulin; PO, per oral; rRT‐PCR, real‐time reverse transcription polymerase chain reaction; TID, three times daily; TPE, therapeutic plasma exchange.

## DISCUSSION

A systematic review covering severe and life‐threatening mucocutaneous eruptions related to COVID‐19 until October 5, 2020, detailed one case of SJS^5^ (Davoodi et al.), one case of SJS‐TEN overlap^6^ (Lagziel et al.) and one case of TEN^7^ (Saha et al.).^8^ Our updated review adds to the literature by describing 31 additional cases up to August 2022, discussing current theories on post‐COVID‐19 infection/vaccination SJS/TEN pathogenesis and comparing treatment strategies.

The pathogenesis of SJS/TEN in the setting of COVID‐19 vaccination is not fully understood, with several theories proposed. Multiple authors have hypothesized that the virotope antigens delivered by COVID‐19 vaccines are expressed on the surfaces of keratinocytes and trigger the activation of cytotoxic CD8+ T‐cells.^2^, ^9^, ^10^, ^11^, ^12^, ^13^ These activated T‐cells then release granulysin, granzyme B, and perforin, which induce pore formation, apoptotic cascades in keratinocytes, and detachment at the dermo‐epidermal junction (DEJ).^2^, ^11^, ^12^ Moreover, these T‐cells secrete significant amounts of cytokines including tumour necrosis factor‐ɑ (TNF‐ɑ) and interferon‐ɣ (IFN‐ɣ), which lead to the overexpression of Fas ligands that also mediate keratinocyte apoptosis.^2^ Certain individuals are more genetically susceptible to the Type IV hypersensitivity reaction, triggering post‐vaccination TEN/SJS as virotope antigens are preferentially expressed on the surfaces of their keratinocytes.^9^ However, others contend that the pathophysiology of post‐COVID‐19 vaccine TEN/SJS lies in the activation of type 1 helper T‐cells (Th1s), which released cytokines such as TNF‐ɑ and IFN‐ɣ that subsequently activate cytotoxic CD8+ T‐cells responsible for triggering keratinocyte apoptosis.^11^, ^13^ The inactive components of COVID‐19 vaccines, or excipients, might also contribute to triggering the type IV hypersensitivity reaction mediating TEN/SJS.^14^, ^15^ Finally, it is hypothesized that the mucosal damage seen in post‐vaccination TEN/SJS is mediated by the host's production of neutralizing antibodies against the SARS‐CoV‐2 spike protein.^16^

Regarding TEN/SJS after COVID‐19 infection, it is hypothesized that the generalized immune activation and the associated increase in cytokines induced by COVID‐19 infection can directly trigger or make individuals more susceptible to TEN/SJS‐inducing type IV hypersensitivity reactions.^17^, ^18^, ^19^ This phenomenon was previously observed in HIV patients and other immunocompromised populations, and some hypothesize that post‐COVID‐19 infection TEN/SJS is a novel example of immune reconstitution inflammatory syndrome (IRIS).^19^

In 17 of the 34 published cases (50%), there were comorbidities identified that included carcinomas, influenza and use of antibiotics, hydroxychloroquine, acetaminophen, non‐steroidal anti‐inflammatory drugs (NSAIDs), chemotherapy and antiepileptics. These findings may constitute a limitation that challenges the association between TEN/SJS and COVID‐19 infection/vaccination. However, COVID‐19 infection may induce immune activation and an inflammatory environment that predisposes patients to drug‐induced TEN/SJS.^18^, ^20^, ^21^ COVID‐19 infections can exacerbate existing drug‐induced TEN/SJS symptoms.^22^

Treatments for post‐infection and post‐vaccination TEN/SJS varied significantly. Cyclosporine is a treatment option that addresses both TEN/SJS and COVID‐19.^17^, ^21^, ^23^ By inhibiting the calcineurin inflammatory pathway, cyclosporine can treat TEN/SJS by inducing immunosuppression; furthermore, cyclosporine targets cyclophilins to inhibit viral replication.^17^, ^21^, ^23^ Cyclosporine was also associated with significantly reduced mortality rates in both COVID‐19 and TEN patients in retrospective studies and has rarely been associated with infection as an adverse effect.^17^, ^23^ Etanercept, a TNF‐ɑ inhibitor, is another option that demonstrated significant oedema reduction, rapid cessation of disease progression and greater reduction of TNF‐ɑ and granulysin expression levels relative to systemic corticosteroids.^2^, ^22^ Etanercept also demonstrated lower rates of gastrointestinal haemorrhage and higher efficacy in promoting re‐epithelialization compared to corticosteroids.^2^ Intravenous immunoglobulin (IVIG) can reduce the severity of COVID‐19 and TEN/SJS by inhibiting T‐cell activation, IL‐6 and TNF‐ɑ while neutralizing exogenous antigens.^7^, ^22^, ^24^ Finally, plasmapheresis is a treatment method for COVID‐19 and TEN/SJS through the removal of serum cytokines and offending drugs/drug metabolites, highlighted by efficacy in patients refractory to systemic corticosteroid therapy.^22^, ^24^ However, others contend that plasmapheresis failed to demonstrate greater benefits relative to other supportive care strategies in a retrospective study.^2^

## CONCLUSIONS

We present an updated review that covers 34 cases of TEN, SJS‐TEN and SJS after COVID‐19 infection and vaccination. Hypotheses for post‐vaccine TEN/SJS onset include virotopes or excipients activating the Th1 pathway, cytotoxic CD8+ T‐cells or cytokines to induce keratinocyte apoptosis. Furthermore, it is hypothesized that COVID‐19 infection can induce immune activation that directly triggers TEN/SJS or increases susceptibility to drug‐induced TEN/SJS. A variety of treatment methods have been promoted as the optimal option, including cyclosporine, etanercept, IVIG and plasmapheresis. We recommend that clinicians remain vigilant for this rare and severe potential complication of COVID‐19 infection and vaccination, as TEN/SJS represents one of the few scenarios where further vaccine doses are contraindicated.

## CONFLICT OF INTEREST

The authors have no conflict of interest to declare.

## ETHICS APPROVAL

IRB approval status: Exempt.
